# Recombinant human IL-26 facilitates the innate immune response to endotoxin in the bronchoalveolar space of mice *in vivo*

**DOI:** 10.1371/journal.pone.0188909

**Published:** 2017-12-05

**Authors:** Aihua Bao, Karlhans Fru Che, Steven Bozinovski, Jie Ji, Joshua A. Gregory, Susanna Kumlien Georén, Mikael Adner, Lars-Olaf Cardell, Anders Lindén

**Affiliations:** 1 Unit for Lung and Airway Research, Institute of Environmental Medicine, Karolinska Institutet, Stockholm, Sweden; 2 Department of Respiratory Medicine, Shanghai General Hospital, School of Medicine, Shanghai Jiao Tong University, Shanghai, P. R. China; 3 RMIT University, School of Health and Biomedical Sciences, Bundoora, Victoria, Australia; 4 Centre for Allergy Research, Karolinska Institutet, Stockholm, Sweden; 5 Unit for Experimental Asthma and Allergy Research, Institute of Environmental Medicine, Stockholm, Sweden; 6 Division of ENT Diseases, CLINTEC, Karolinska Institutet, Stockholm, Sweden; 7 Lung Allergy Clinic, Karolinska University Hospital, Stockholm, Sweden; Katholieke Universiteit Leuven Rega Institute for Medical Research, BELGIUM

## Abstract

Interleukin (IL)-26 is released in response to bacterial endotoxin (LPS) in the bronchoalveolar space of humans *in vivo* and it may potentiate neutrophil chemotaxis by enhanced IL-26 receptor stimulation. However, the effects of extracellular IL-26 protein on the innate immune response in the lungs *in vivo* remain unknown. Here, we characterized these effects of IL-26 on a wide range of aspects of the innate immune response to LPS in different compartments of the lungs *in vivo* over time. We administrated recombinant human (rh) IL-26 protein in the bronchoalveolar space using intranasal instillation in a mouse *in vivo* model, with and without prior instillation of LPS. We verified gene expression of the IL-26 receptor complex in mouse lungs and observed that, after instillation of LPS, rhIL-26 increases the phosphorylation of STAT3, a signaling molecule of the IL-26 receptor complex. We also observed that rhIL-26 exerted additional stimulatory and inhibitory actions that are compartment- and time-dependent, resulting in alterations of cytokines, proteinases, tissue inflammation and the accumulation of innate effector cells. Without the prior instillation of LPS, rhIL-26 exerted time-dependent effects on total gelatinase activity but few other effects. Most important, after instillation of LPS, rhIL-26 cleared inflammatory cells from local tissue and increased the accumulation of innate effector cells in the bronchoalveolar space. Tentatively, rhIL-26 may facilitate the innate immune response towards the bronchoalveolar space *in vivo* and represents a potential target for therapy in lung disorders involving the innate immune response.

## Introduction

Bacterial infections in the lungs remain important causes of morbidity and mortality world-wide [1]. The logistical burden of these infections and the related resistance against antibiotics constitutes a major problem for health care at the global level. Thus, there is an unmet need for new and effective therapeutic strategies targeting the innate immune response in the lungs. To establish a rationale for these, there is a corresponding need to improve the understanding of the immunological mechanisms controlling this innate immune response [1].

The cytokine interleukin (IL)-26 belongs to the IL-10 family but it is also positioned as a Th17-associated cytokine [2]. Given that Th17 lymphocytes are involved in mediating the innate immune response in mammalian lungs, it seems logical that IL-26 is released in response to bacterial endotoxin (lipopolysaccharide, LPS) in the bronchoalveolar space of humans [3,4]. However, in this pulmonary compartment, IL-26 is expressed not only in Th17 cells but also in other lymphocytes and in macrophages. Moreover, this cytokine is substantially more abundant in the bronchoalveolar space than is the archetype Th17 cytokine IL-17A [4] and its mechanisms of action may be unique [5,6,7,8,9]. In this context, the potentiation of the chemotactic response of human blood neutrophils by recombinant human (rh) IL-26 protein *in vitro* here stands out as particularly interesting [4]. This potentiation is likely to be mediated by the IL-26 receptor complex; a receptor complex that is present in the cell membrane of human neutrophils and may respond to rhIL-26 with altered phosphorylation and gene expression of the intracellular signaling molecule STAT3 [4]. Importantly, the integrative effects of the referred receptor stimulation on the innate immune response in the bronchoalveolar space *in vivo* have remained unknown until date, possibly due to the absence of a known orthologue to IL-26 in translational animal models including the mouse model [10].

When evaluating the utility of the mouse model for the study of effects caused by IL-26, it is important to understand that the two sub-units of the IL-26 receptor complex are present in mice and specifically so in the lungs [11,12,13]. Thus, even though the mouse genome does not contain the coding sequence for IL-26 protein *per se*, it does contain the coding sequence of both sub-units of the IL-26 receptor complex [11,12,13]. Moreover, the IL-26 receptor complex mediates intracellular signaling in airway epithelial cells from mice [11,12,13]. In line with this, transgenic over-expression of human IL-26 in mouse lymphocytes enhances the production of collagen in the lungs *in vivo* and a mouse fibroblast cell line responds with enhanced collagen production to rhIL-26 *in vitro* [14]. Given these fundamental observations, we hypothesized that enhanced IL-26 receptor stimulation by the “model agonist” rhIL-26 in the bronchoalveolar space will cause relevant immunological effects over time in mice *in vivo*. Thus, by utilizing this animal model, we may reveal down-stream effects of IL-26 receptor stimulation on key aspects of the control of the innate immune response in mammalian lungs, such as induced alterations of cytokines, proteinases, tissue inflammation and the accumulation of innate effector cells (i.e. macrophages and neutrophils). The current study aimed to evaluate this hypothesis and we did this with and without prior activation of the innate immune response to LPS in the bronchoalveolar space, since it has previously been shown that local IL-26 is released in this compartment after this stimulation of TLR-4 in humans [4].

## Material and methods

### Animals

Male Balb/c mice (18–20 g) were obtained from the Harlan Sprague-Dawley Inc. (Leiden, Netherlands) and were housed under specific pathogen-free conditions with food and water *ad libitum* under careful monitoring. The study protocol was approved by Regional Committee of Animal Experimentation Ethics in Stockholm, Sweden (Diary No. N258-13).

### Instillation of rhIL-26 with or without prior instillation of LPS

After light anesthesia with isoflurane, the mice received intranasal instillation of carrier-free rhIL-26 dimer protein (1 μg/mouse; R&D Systems Inc.^™^, Abingdon, UK) dissolved in sterilized phosphate-buffered saline (PBS, 40μL), using technical procedures as previously described [15]. The control mice received PBS (vehicle) only instead of LPS. This instillation of rhIL-26 was done with or without prior instillation of LPS (10 mg/kg: Escherichia coli 0127:B8, Sigma-Aldrich Co. LLC., St. Louis, MO) or its vehicle (sterilized PBS), with a 10 minute separation of these two instillations, resulting in 4 treatment groups at each time-point of harvest. Control mice received PBS (vehicle) only instead of rhIL-26. The chosen dose of LPS and rhIL-26, respectively, was the most efficacious among three doses of LPS (0.5, 1 & 10 mg/kg) and two doses of rhIL-26 (0.1 & 1 μg/mouse), using BAL leukocyte concentrations as outcome parameter for comparative effects. The mice were euthanized by using a lethal dose of pentobarbital (100 mg/kg i.p.) at three different time-points (6, 24 & 72 h) post treatment and samples (BAL & lung tissue) were harvested.

### Processing and leukocyte differential counts for BAL samples

Bronchoalveolar lavage (BAL) was performed utilizing euthanized mice as previously described [16]. In brief, after tracheal intubation, the mice were instilled with sterile cold PBS (0.8 mL) and flushed (×3). The total BAL leukocyte concentration was determined immediately using a hemocytometer. After centrifugation (1000 ×g; 10 min, 4°C), the cell-free BAL fluid was then aliquoted and stored (−80°C) for further analyses (see below). The cell pellet was then re-suspended in PBS and underwent careful centrifugation (600 rpm, 4 min) in a designated centrifuge (StatSpin^®^ Cytofuge 2, Beckman Coulter Inc,^™^, Indianapolis, IN) The cytospin slides were then stained (May-Grunwald- Giemsa^™^, Sigma-Aldrich Co. LLC., MO) and the cell differential counts were performed in a blinded manner by an experienced operator. The operator counted 300 leukocytes (i.e. macrophages, neutrophils & lymphocytes) per slide, all identified by standard morphology using light microscopy (×400 magnification; Olympus Optical^™^, Tokyo, Japan).

### Cytokine protein concentrations in BAL samples

The concentrations of cytokine proteins in cell-free BAL fluid (CXCL2 [MIP-2], CXCL1 [KC], CCL3 [MIP-1α], CCL2 [MCP-1], G-CSF, IL-6, TNF-α) were quantified utilizing Luminex^™^ (Bio-Plex 200^®^, Bio-Rad Laboratories, Inc.^™^, CA) and the Bio-Rad mouse cytokine kit (Bio-Plex cytokine assay^®^, Bio-Rad Laboratories, Inc.^™^, CA) according to the manufacturer’s instructions. Data were collected with a minimum of 100 beads *per* analyte utilizing the Bio-Plex Manager Software (Bio-Rad Laboratories, Inc.^™^, CA).

### Processing of and scoring of inflammation in lung tissue samples

After the BAL procedure, the left lung lobe was removed, placed in neutral-buffered formalin solution (10%) and embedded in paraffin. The remaining lobes were micro-dissected and then kept frozen (-80°C) for protein extraction or in RNAlater^®^ Stabilization Solution (Life Technologies^™^, CA) for RNA extraction (-20°C).

The hematoxylin and eosin (H&E) staining were performed on five-micrometer thin paraffin sections after de-paraffinization and rehydration as previously described [16].

To evaluate the spatial alterations of infiltrated innate effector cells in lung tissues, four different pulmonary compartments were defined in tissue sections stained with H&E as follows: the interstitial space, the alveolar space, the peribronchial area and the perivascular area. A scoring system was adopted from a protocol described by the American Thoracic Society [17], where a value of 0 was recorded when no inflammatory cells were found; a value of 1 for 5 or less inflammatory cells, and a value of 2 for more than 5 inflammatory cells in the interstitial space and the alveolar space; for the peribronchial and the perivascular areas, a value of 1 represents sporadic inflammatory cells, and 2 means a thin layer (1–5 cells) and 3 representative of a thick layer (> 5 cells). Slides were coded and 10 random high-power fields (×200) per slide were independently scored by two qualified and independent investigators in a blinded manner using light microscopy (Olympus Optical^™^, Tokyo, Japan). Ten random fields on tissue (1–3 sections, depending upon the access to material) per slide of each mouse were carefully scrutinized. The inflammation scores in each compartment were expressed as a mean value of the referred 10 field counts. The total lung inflammation score was defined as the average of the scores from the four different compartments.

### Phosphorylation of STATs in lung tissue samples

Protein was extracted from lung tissues and investigated by Western blot as previously described [17]. In brief, mouse lung tissues were lysed with ice-cold radioimmunoprecipitation assay (RIPA) buffer (Cell signaling technology, MA) containing a proteinase inhibitor (cOmplete^®^) and a phosphatase inhibitor (PhosSTOP^®^) (both from Roche Diagnostics Co.^™^, IN) and homogenized (Precellys^™^, Rockville, MD). After centrifugation (15,000 ×g; 15 min; 4°C, Sorvall Legend Micro 21R, Life Technology Inc^™^, CA), the protein was quantified and subjected to the subsequent SDS—PAGE (10%).

After electrophoresis and transfer, the membranes were blocked with Bovine Serum Albumin (1%, in TBST [20 mM Tris pH 7.5. 150 mM NaCl. 0.1% Tween 20]) overnight (4°C). The binding of the primary antibody was detected by infrared dye-conjugated secondary antibodies and the Odyssey CLx system (both from Li-Cor Inc.^™^, NE). The primary antibodies (rabbit anti-mouse) were as follow: mouse β-Actin (1:500), STAT1/phospho-STAT1 (1:1000) and STAT3/phospho-STAT3 (1:1000) antibodies (all from Cell Signaling Technology Inc.^™^, MA), and myeloperoxidase (MPO) heavy chain (1:200; Santa Cruz Biotechnology Inc.^™^., Texas).

### Messenger RNA for cytokines and sub-units of the IL-26 receptor complex in lung tissue samples

Real-time reverse transcription polymerase chain reaction (RT-PCR) was performed as previously described [18]. In brief, mRNA was prepared from homogenized tissues using a commercial Spin technology (RNeasy Mini^®^, Qiagen^™^, MD) according to the manufacturer’s instructions and the purity verified using Nanodrop^®^ 2000 (Life Technology Inc ^™^, CA). First-strand cDNA was made from mRNA (2 μg) in a reaction volume (20 μL) using the high capacity RNA-cDNA kit (Life Technology Inc^™^, CA) according to instructions. Real time PCR was then performed using the Prism 7500HT instruments, (Life Technology Inc^™^, CA). Each sample was run in duplicates in a reaction mixture (10 μL) that contained (5 pmol/μL) of forward and reverse primer (1 μL each), Fast SYBR Green^®^ Master Mix (5 μL, Life Technology Inc^™^, CA), templates cDNA (4 ng in 2 μL) and water (1 μL). The sequences of murine forward and reverse primers used are displayed in Table 1 (synthesized by Cybergene^™^ AB, Solna, Sweden). The thermal cycling program of qPCR was set in accordance with the manufacturer’s instructions and the relative gene expression was normalized to β-actin. Data were expressed as fold-increase in mRNA expression compared with one of the control animals, that was attributed a value of 1.

**Table 1:** Primer sequences.

Primer	Forward primer	Reverse primer
ACTIN	5′- GGTGGGAATGGGTCAGAAGG-3′	5’- GGGGTACTTCAGGGTCAGGA -3’
CXCL1	5′-ACCGAAGTCATAGCCACACT-3′	5′- GTGCCATCAGAGCAGTCTGT -3′
TNF-α	5′- TAGCCCACGTCGTAGCAAAC -3′	5′- ACCCTGAGCCATAATCCCCT -3′
CCL20	5′- ATCTGTGTGCGCTGATCCAA-3′	5′-CTTGACTCTTAGGCTGAGGAGG-3′
IL-6	5′- TTCCTCTCTGCAAGAGACTTCC -3′	5′- AGTCTCCTCTCCGGACTTGT-3′
G-CSF	5′- CATGAAGCTAATGGCCCTGC -3′	5′- GGGGTGACACAGCTTGTAGG -3′
CCL2	5′- GCTGTAGTTTTTGTCACCAAGC -3′	5′- AAGGCATCACAGTCCGAGTC -3′
CXCL2	5′- CAGGCTACAGGGGCTGTTGT-3′	5′- ACATCAGGTACGATCCAGGC-3′
IL-10R2	5′-CCTTCTGGTGCCAGCTCTAGG-3′	5′-GTGACATTGACCCACTCCGA-3′
IL-20R1	5′-AGAGGTTGCCCTGACAACTG-3′	5′-CACTGGGACCACGTTCTTCT-3′

### Total proteinase activity in BAL samples

Zymography was used to assess total proteinase activity (anti-proteinases dislodged from proteinases), including that of the macrophage gelatinase Matrix metalloproteinase (MMP)-2 [19] and the macrophage and neutrophil gelatinase MMP-9 [20], as previously described [21]. Briefly, cell-free BAL fluid samples of each group were pooled (i.e. samples from 8 mice pooled into 1 loading sample and run in duplicate), concentrated, spun and the pellet washed and re-suspended in non-reducing buffer (50 μL; 1×). Of this non-reducing buffer, 20 μL was loaded on SDS-PAGE mini-gels (10%) containing gelatin (2 mg/mL) and run at a constant voltage (200 V; 45 min; Mini-PROTEAN^®^, Bio-Rad Laboratories, Inc., CA). The gels were then washed in Triton X-100^™^ (2.5%), incubated overnight in zymography buffer (37°C), stained with Coomassie Brilliant Blue R-250 (45 min, from Sigma-Aldrich Co. LLC., MO) and subsequently extensively distained.

### Net gelatinase and serine proteinase activity in BAL samples

These measurements were based upon functional substrate assays, described in detail elsewhere [22]. Briefly, the *net* gelatinase activity in cell-free BAL fluid was quantified to functionally assess excess proteolytic activity generated by gelatinases. Here, the specific fluorescence conjugated gelatin substrate (EnzChek^®^, Molecular Probes^™^, Queensland, Australia) was incubated with cell-free BAL fluid (room temperature, 2 h). The fluorescence intensity of the digested substrate was measured in a microplate reader (Victor II^™^, Wallac, Melbourne, Australia) to detect quantitative differences in activity and the result was expressed in arbitrary units. The *net* serine proteinase activity was measured utilizing a specific fluorescence conjugated elastin substrate (EnzChek^®^, Molecular Probes^™^, Queensland, Australia), diluted in a reaction buffer and incubated with cell-free BAL fluid (room temperature, 2 h) [23]. The fluorescence of digested substrate was detected by a microplate reader (emission detection at 515 nm) and the results were expressed in arbitrary units.

### Extracellular and intracellular concentrations of proteinase proteins in BAL samples

The extracellular concentrations of proteinase protein were measured in cell-free BAL samples utilizing commercial ELISA kits for MMP-9 (detecting pro and active as well as TIMP-bound forms), pro-MMP-9 (pro-MMP-9 only), MMP-2 (detecting pro and active as well as TIMP-bound forms), myeloperoxidase (MPO) and neutrophil elastase (NE) (R&D Systems, Inc., MN.); cathepsin G (MyBioSource Inc.^™^, CA.) in accordance with the manufacturer’s instructions. The intracellular proteinases (except for cathepsin G) were measured in the BAL cells lysate using ELISA as well. In brief, the BAL cell pellets of each group were lysed in the RIPA buffer with gentle shaking (4°C, 30 min), centrifuge (1,500 × g, 3 min) and the supernatant was then analyzed. The results were standardized to volume and expressed as cellular content of proteinases (per 1,000 cells).

### Statistics

Normal distribution was ascertained for all data sets after data was sorted into study groups. We present these data as mean with SEM where n represents the number of independent observations. Differences were determined by one-way analysis of variance (ANOVA) followed by Fisher’s protected least significant difference (PLSD) post hoc test for multiple comparisons, where appropriate. The bivariate (Pearson) correlation test was used as appropriate. All statistical analyses were performed using the computer software SPSS 17.0. GraphPad Prism^™^ for Windows was utilized for figures conformation (Version 4.02, GraphPad Software Inc., CA.). A p-value less than 0.05 was required for statistical significance.

## Results

### Effects of rhIL-26 on STAT phosphorylation in lung tissue

**Fig 1:**
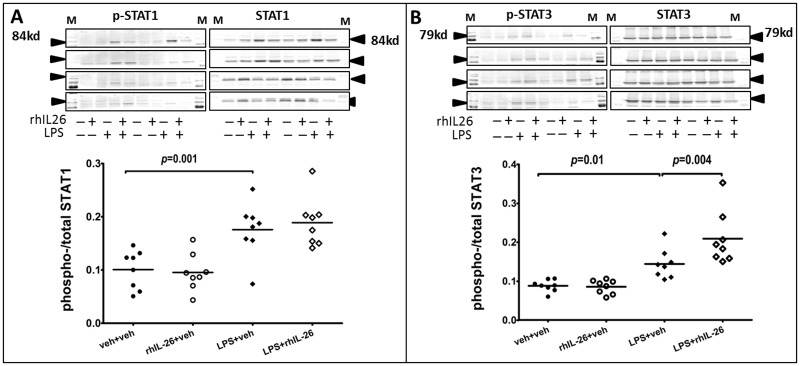
Effects of rhIL-26 on STATs phosphorylation in lung tissue. Mice received intranasal instillation of recombinant human (rh) IL-26 protein or its vechicle (PBS), with or without prior instillation of endotoxin (LPS) or its vehicle (PBS). The total and phosphorylated STAT1 **(A)** and STAT3 **(B)** proteins were measured with Western blot in lung tissues 24 hours (h) after the instillations (n = 10). Each lane of the bands represents a separate blot. Data are presented as mean ± SEM.

The prior intranasal instillation of LPS alone, without the subsequent instillation of rhIL-26, enhanced the phosphorylation of both STAT1 and STAT3 in lung tissue samples harvested at the intermediate 24 h time-point. The subsequent intranasal instillation of rhIL-26, after that of LPS, clearly enhanced the phosphorylation of STAT3 but did not exert any reproducible effect on not that of STAT1 (Fig 1A and 1B). The instillation of rhIL-26 alone, without the prior instillation of LPS, elicited no reproducible effects on the phosphorylation of either STAT1 or STAT3.

To confirm previous reports on the presence of the IL-26 receptor complex in the mouse model [11,12,13], we examined the gene expression (mRNA) of the IL-26 receptor sub-units IL-10R2 and IL-20R1 in lung tissue, at the early 6 h and intermediate 24 h time-point after instillation of rhIL-26, with and without prior instillation of LPS. Here, the mRNA of each receptor sub-unit was clearly detectable at both referred time-points but neither the instillation of rhIL-26 nor that of LPS, alone or in combination, altered the gene expression of these receptor sub-units in a reproducible manner (S1 Fig).

### Effects of rhIL-26 on the accumulation of BAL cells and cytokines

**Fig 2:**
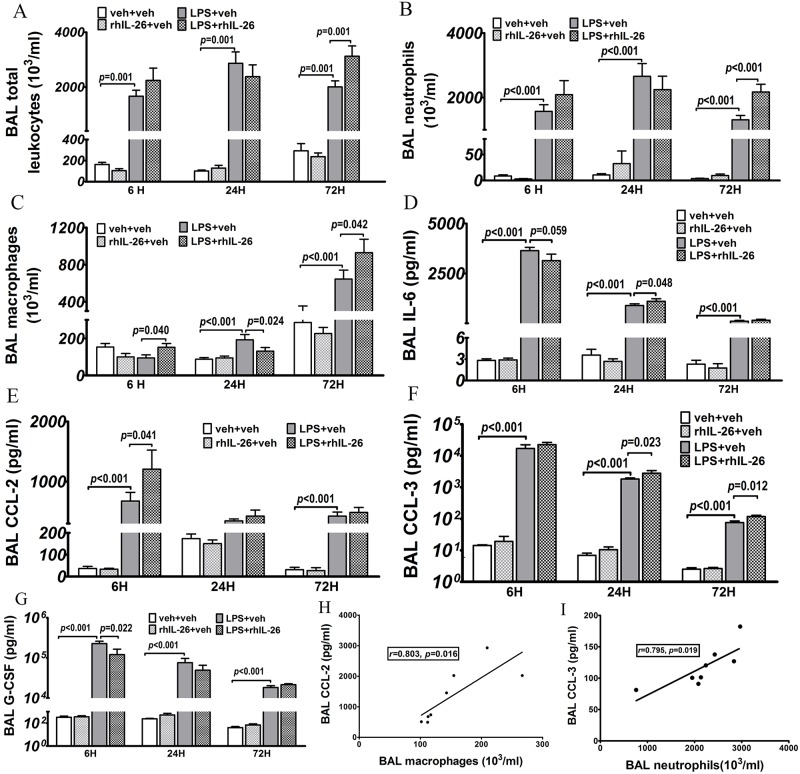
Effects of rhIL-26 on the accumulation of BAL cells and cytokines. Mice received intranasal instillation of recombinant human (rh) IL-26 protein or its vechicle (PBS), with or without prior instillation of endotoxin (LPS) or its vehicle (PBS). Bronchoalveolar lavage (BAL) samples were harvested 6 hours (h) (n = 8), 24 h (n = 10) and 72 h (n = 8) after the instillations. The number of **(A)** total leukocytes and differential cellularity including **(B)** neutrophils, **(C)** macrophages and lymphocytes (S1 Table) were determined. Cytokines were measured using Luminex^™^ in the cell-free BAL fluid for **(D)** IL-6, **(E)** CCL2, **(F)** CCL3 and **(G)** G-CSF. Correlation between concentrations of **(H)** CCL2 protein and macrophages in 6 h BAL samples and between concentrations of **(I)** CCL3 and neutrophils in 72 h BAL samples were also calculated based upon the acquired data. Data are presented as mean ± SEM.

The initial instillation of LPS alone, without the subsequent instillation of rhIL-26, did substantially increase the total leukocyte and neutrophil concentrations in BAL samples at the early 6 h, intermediate 24 h and late 72 h time-points (Fig 2A and 2B). However, after the initial instillation of LPS, the BAL macrophage concentrations were enhanced only at the intermediate 24 h and the late 72 h time-point (Fig 2C). The lymphocyte concentrations in BAL samples were very low at all-time-points, irrespectively of study group (S1 Table).

The subsequent instillation of rhIL-26, after that of LPS, clearly altered BAL macrophage and neutrophil concentrations, the nature of which depended upon the specific time-point of sampling (Fig 2A, 2B and 2C). Thus, this subsequent instillation of rhIL-26 enhanced BAL macrophage concentrations at the early 6 h time-point and caused a corresponding decrease at the intermediate 24 h time-point. Moreover, this subsequent instillation of rhIL-26 caused a clear enhancement of the total BAL leukocyte as well as macrophage and neutrophil concentrations but only at the late 72 h time-point (Fig 2A, 2B and 2C). The instillation of rhIL-26 alone did not cause any pronounced alteration in BAL leukocyte concentrations at either time-point.

The mouse orthologue of IL-26 remains unknown [10]. Given this, we addressed how stimulation with rhIL-26may contribute to the mobilization of innate effector cells in the lungs by quantifying cytokine proteins. Thus, we did this in mice instilled with LPS with and without the subsequent instillation of rhIL-26. Specifically, we targeted the pro-inflammatory cytokines IL-6 and TNF-α, the neutrophil-mobilizing chemokines CXCL1 and CXCL2, the macrophage-mobilizing chemokines CCL2 and CCL3 and the granulocyte growth factor G-CSF in cell-free BAL fluid samples using Luminex^™^, in principle a multi-ELISA approach. Our results showed substantially increased protein concentrations of these cytokines at all three time-points after instillation of LPS alone (Fig 2D, 2E, 2F and 2G & S2 Fig). Moreover, our results revealed that the subsequent instillation of rhIL-26 enhanced the concentrations of CCL2 at the early 6 h time-point (Fig 2E) whereas rhIL-26 decreased the concentrations of G-CSF at the same time-point (Fig 2G). In addition, this subsequent instillation of rhIL-26 after that of LPS did increase IL-6 and CCL3 concentrations at the intermediate 24 h time-point (Fig 2D and 2F) and the same was true for CCL3 at the late 72 h time-point (Fig 2F).

We further addressed the correlation between the concentrations of cytokines and their most likely leukocyte sources in mice after prior instillation of LPS followed by subsequent instillation of rhIL-26. Here, we detected a strong positive correlation between the concentrations of CCL2 and macrophages at the early 6 h time-point. Similarly, we detected a strong positive correlation between CCL3 and neutrophil concentrations at the late 72 h time-point (Fig 2H and 2I).

### Effects of rhIL-26 on mRNA for cytokines and sub-units of the IL-26 receptor complex in lung tissue samples

**Fig 3:**
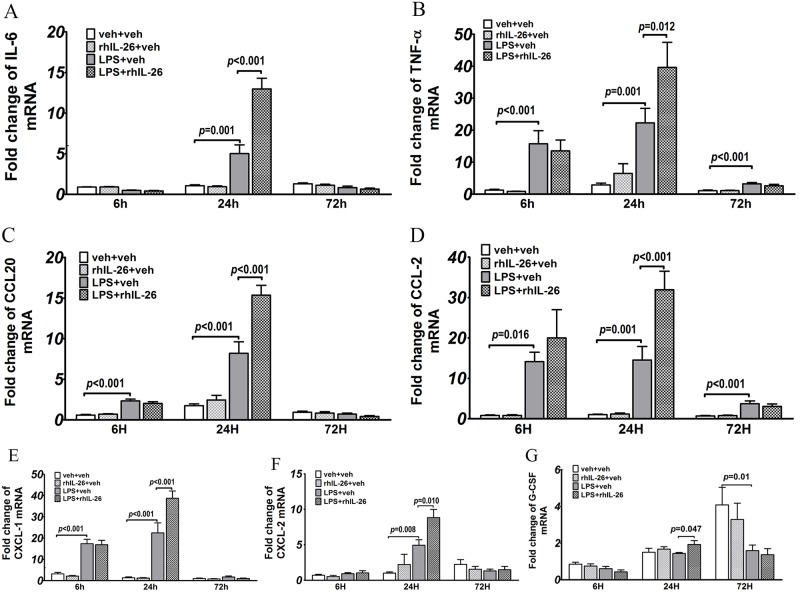
Effects of rhIL-26 on cytokine mRNA in lung tissue. Mice received intranasal instillation of recombinant human (rh) IL-26 protein or its vechicle (PBS), with or without prior instillation of endotoxin (LPS) or its vehicle (PBS). Lung tissue samples were harvested 6 h (n = 8), 24 h (n = 10) and 72 h (n = 8) after the instillations. The harvested tissue was assessed for messenger (m) RNA of cytokines using RT-qPCR. Data shown includes **(A)** IL-6, **(B)** TNF-α, **(C)** CCL20, **(D)** CCL2, (E) CXCL1, **(F)** CXCL2 and **(G)** G-CSF. Data are presented as mean ± SEM.

We also characterized the levels of mRNA matching the targeted cytokine proteins (see above) in lung tissue samples using RT-PCR (Fig 3A, 3B, 3C, 3D, 3E, 3F and 3G). Here, the initial instillation of LPS alone enhanced the mRNA levels for IL-6 (24 h), TNF-α (6, 24 & 72 h), CCL20 (6 & 24 h), CCL2 (6, 24 & 72 h), CXCL1 (6 & 24 h) and CXCL2 (24 h) whereas the mRNA levels for G-CSF were down-regulated (72 h). The subsequent instillation of rhIL-26, after the prior instillation of LPS, caused a clear additional enhancement of mRNA levels for IL-6, TNF-α, CCL20, CCL2, CXCL1, CXCL2 and G-CSF at the intermediate 24 h time-point only (Fig 3A, 3B, 3C, 3D, 3E, 3F and 3G). However, the instillation of rhIL-26 alone did not cause any substantial effect on the mRNA levels at either time-point (Fig 3A, 3B, 3C, 3D, 3E, 3F and 3G).

### Effects of rhIL-26 on lung tissue inflammation and MPO protein content

**Fig 4:**
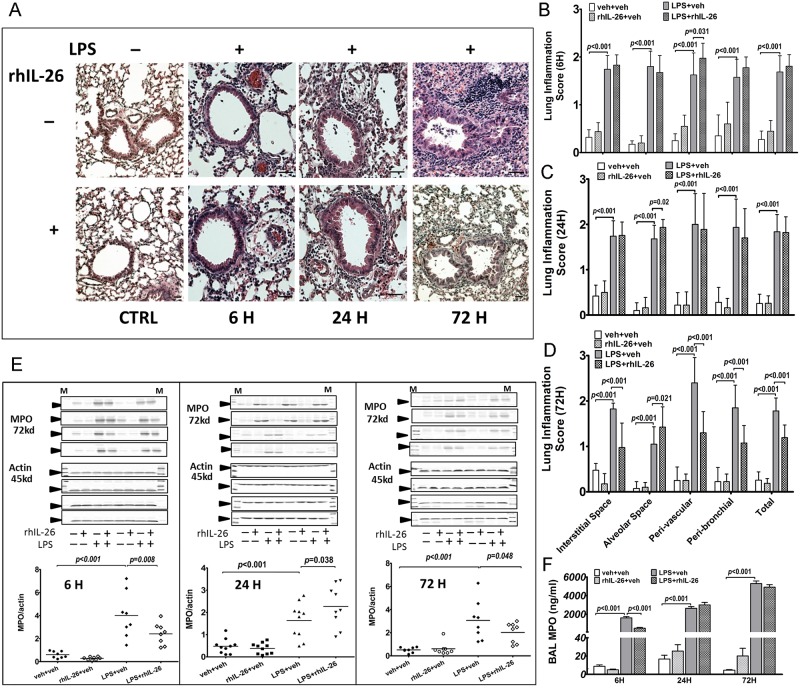
Effects of rhIL-26 on lung tissue inflammation and MPO protein content. Mice received intranasal instillation of recombinant human (rh) IL-26 protein or its vechicle (PBS), with or without prior instillation of endotoxin (LPS) or its vehicle (PBS). Lung tissue samples were harvested 6 h (n = 8), 24 h (n = 10) and 72 h (n = 8) instillations. Histological evaluations were performed on lung tissue sections stained with hematoxylin and eosin (H&E) and quantified with a scoring system. Representative H&E-staining pictures (magnification: 10×, scale bar: 50 μm) are shown **(A)**. The inflammation in four tissue compartments (the interstitial area, alveolar space, peribronchial area and perivascular area) on lung tissue sections was evaluated and scored (see Methods section) at **(B)** 6 h, **(C)** 24 h, and **(D)** 72 h after the intranasal instillation, respectively. The MPO protein in lung tissue was analyzed using immunoblot. The bands of each mouse and the densitometry ratio of MPO to actin are shown in **(E)**. Each panel relates to a time-point and each lane of the bands represents a separate blot. **(F)** The MPO content in cell-free BAL fluid samples was quantified using ELISA. Data are presented as mean ± SEM.

We characterized the effects of the instillation of rhIL-26 on lung tissue inflammation after the instillation of LPS in four (4) different pulmonary compartments (Fig 4A). These compartments included the interstitial lung space, the bronchoalveolar space, the perivascular lung area and the peribronchial lung area. After the intranasal instillation of LPS alone, we observed high scores of inflammation in all 4 compartments, at all time-points (Fig 4B, 4C and 4D). Here, the addition of the subsequent instillation of rhIL-26 further enhanced the inflammation score in the perivascular area at the early 6 h time-point (Fig 4B) and in the bronchoalveolar space at the intermediate 24 h time-point (Fig 4C). After this instillation of rhIL-26, there was a consistent decrease in the inflammation score in the interstitial space, the perivascular and the peribronchial area at the late 72 h time-point (Fig 4D). However, the latter was not the case in the alveolar space, where the inflammation score was increased (Fig 4D).

Given that rhIL-26 caused compartment- and time-related alterations of inflammatory cells in lung tissue after instillation of LPS, we examined whether these induced alterations are associated with neutrophil activity assessed as MPO protein [24,25]. In terms of MPO, the initial instillation of LPS enhanced its protein content in lung tissue (Fig 4E) at all time points (6, 24 & 48 h). Here, the subsequent instillation of rhIL-26 altered the MPO protein content in lung tissue in a time-dependent manner. Thus, the MPO protein content was decreased by the subsequent instillation rhIL-26 at the early 6 h and the late 72 h time-point, respectively, whereas it was increased at the intermediate 24 h time-point (Fig 4E).

To further characterize the impact of rhIL-26 on MPO in the lungs, we also measured the concentrations of MPO protein in cell-free BAL fluid samples. Here, the instillation of LPS significantly enhanced MPO protein concentrations in these BAL fluid samples at the early 6 h and the late 72 h time-point, respectively (Fig 4F). The subsequent instillation of rhIL-26 clearly decreased the concentration of MPO protein in BAL fluid samples at the early 6 h time-point but caused no substantial effects at the intermediate 24 h and the late 72 h time-point, respectively (Fig 4F).

### Effects of rhIL-26 on the activity and quantity of proteinases in BAL samples

**Fig 5:**
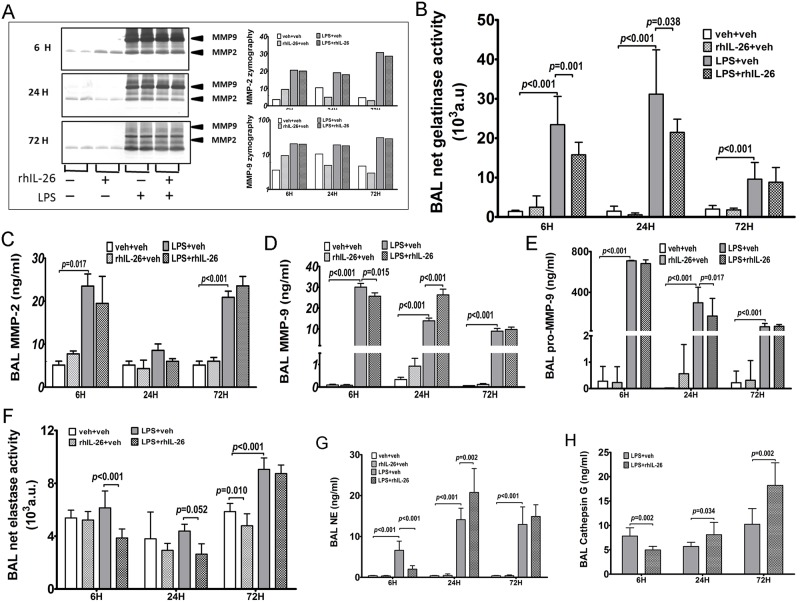
Effects of rhIL-26 on the activity and the quantity of proteinases in BAL samples. Mice received intranasal instillation of recombinant human (rh) IL-26 protein, or its vehicle (PBS), with or without prior instillation of endotoxin (LPS). Bronchoalveolar lavage (BAL) samples were harvested 6 h (n = 8), 24 h (n = 10) and 72 h (n = 8) after instillations. The total activity of gelatinase (A) was measured with zymography in BAL samples that were pooled for each study group: the left panel presents the bands for MMP-9 and MMP-2 detected at all three time-points and the right panel shows the bands densitometry data of MMP-2 (upwards) and MMP-9 (downwards) **(A)**. The respective net activity of **(B)** gelatinase and **(F)** elastase in the same BAL samples was measured utilizing a substrate-based method. The extracellular concentrations of gelatinases including **(C)** MMP-2, **(D)** MMP-9, **(E)** pro- MMP-9, and serine proteinases including **(G)** NE and **(H)** Cathepsin G (only in mice receiving instillation of LPS) were quantified in BAL samples using ELISA. Data are presented as mean.

We also characterized the effects of rhIL-26 on the activity and quantity of proteinases that are released by innate effector cells. We first measured the total activity of gelatinase in cell-free BAL fluid samples with zymography. Here, the densitometry data demonstrated that after the instillation of LPS alone, the quantitatively dominating gelatinases were MMP-2 and MMP-9, with MMP-9 being most abundant (Fig 5A). Furthermore, the average total gelatinolytic activity of both MMP-2 and -9 tended to be enhanced by the initial instillation of LPS alone at all three time-points (Fig 5A).

In mice that received the initial instillation of LPS, the densitometry of zymography gels demonstrated similar levels of total gelatinase activity, both with and without the addition of the subsequent instillation of rhIL-26 (Fig 5A). However, we did observe differences in total gelatinase activity caused by the instillation of rhIL-26 protein alone (Fig 5A). Specifically, the instillation of rhIL-26 alone tended to decrease the total MMP-9 activity at the intermediate 24 h and the late 72 h time-point, respectively (Fig 5A). Furthermore, the instillation of rhIL-26 only tended to increase the total activity of MMP-2 at the early 6 h time-point and to decrease it at the intermediate 24 h time-point.

To clarify the impact of rhIL-26 on the net gelatinase activity (i.e. the activity in the presence of endogenous anti-proteinases), we measured the net gelatinolytic activity in cell-free BAL fluid, utilizing a substrate-based assay. Here, the initial instillation of LPS alone enhanced the net gelatinase activity at all time-points (Fig 5B). Notably, the addition of the subsequent instillation of rhIL-26 did exert a substantial inhibitory effect on the net activity of gelatinases in cell-free BAL sample: an effect that was evident at the early 6 h and the intermediate 24 h time-point, respectively (Fig 5B). However, this particular effect was diminished at the late 72 h time-point.

To determine whether the alterations in proteinase activity relate to quantitative alterations, we measured the protein concentrations of MMP-2 and -9 in cell-free BAL fluid samples utilizing ELISA. Here, the initial instillation of LPS alone increased extracellular (i.e. in the BAL fluid) MMP-2 concentrations at the early 6 and the late 72 h time-point, respectively, whereas no substantial effect of this LPS instillation was observed at the intermediate 24 h time-point (Fig 5C). The addition of the subsequent instillation of rhIL-26 caused no substantial effects at any of the three time-points (Fig 5C).

In contrast to the case for MMP-2 protein, the initial instillation of LPS alone increased extracellular MMP-9 concentrations at all time-points (Fig 5D). Moreover, after the prior instillation of LPS, the subsequent instillation of rhIL-26 did decrease the MMP-9 concentrations at the early 6 h time-point, whereas the same instillation increased the MMP-9 concentrations at the intermediate 24 h time-point (Fig 5D). However, this instillation of rhIL-26 caused no substantial alterations in MMP-9 concentrations at the late 72 h time-point. Of note, the sole instillation of rhIL-26 *per se* caused no substantial effect on MMP-9 concentrations at either of the three time-points.

In addition to “total” MMP-9 (including the pro-, active and TIMP-bound forms), we also selectively measured the pro-form of this enzyme using ELISA. Here, the initial instillation of LPS only increased the concentration of pro-MMP-9 in cell-free BAL fluid of mice at all time-points (Fig 5E). The addition of the subsequent instillation of rhIL-26 decreased the pro-MMP-9 concentrations at the intermediate 24 h but not at the early 6 h nor at the late 72 h time-point, respectively (Fig 5E).

Given that the serine proteinases are major components of proteolytic activity, we also examined the impact of rhIL-26 on the *net* activity of these proteinases in cell-free BAL fluid samples, utilizing a substrate-based assay. Here, the initial instillation of LPS caused an increase in the net serine proteinase activity at the late 72 h time-point but not at the early 6 h or intermediate 24 h time-point (Fig 5F). In the mice receiving prior instillation of LPS, the addition of the subsequent instillation of rhIL-26 clearly decreased the net serine proteinase activity in cell-free BAL fluid samples harvested at the early 6 h time-point; this decrease was more modest at the intermediate 24 h and it diminished at the late 72 h time-point. Of note, intranasal instillation of rhIL-26 alone, thus without the prior instillation of LPS, caused a clear inhibition on the net serine proteinase activity at the late 72 h time-point only (Fig 5F).

In analogy to the reasoning for gelatinases, we quantified extracellular concentrations of neutrophil elastase and cathepsin G in cell-free BAL fluid samples as well. Here, we found that the initial instillation of LPS only increased extracellular concentrations of NE at all time-points (Fig 5G). The addition of the subsequent instillation of rhIL-26 decreased the NE concentrations at the early 6 h time-point whereas, in contrast, this cytokine instillation increased NE concentrations at the intermediate 24 h time-point, without any reproducible effect at the late 72 h time-point (Fig 5G).

Utilizing ELISA, we quantified cathepsin G concentrations in cell-free BAL fluid samples from mice with prior intranasal instillation of LPS, with and without the addition of the subsequent instillation of rhIL-26. Here, the response of cathepsin G to the addition of the subsequent instillation of rhIL-26 was qualitatively similar to that of NE, with a clear inhibitory effect on cathepsin G concentrations at the early 6 h time-point, a moderate increasing effect at the intermediate 24 h time-point and, finally, a more pronounced increasing effect at the late 72 h time-point (Fig 5H).

### Effects of rhIL-26 on extracellular and intracellular proteinases in relation to innate effector cells in BAL samples

To further address the influence of rhIL-26 on proteinases associated with innate effector cells in the bronchoalveolar space, we assessed the quantity of proteinases *per* principal cellular source in BAL samples. We utilized the data on proteinase concentration from ELISA analyses of cell-free BAL fluid and chose the data on the concentration of the most likely leukocyte source from BAL cell differential counts as denominator. With this approach, we thus calculated the extracellular concentration of MMP-2 *per* macrophage, NE and cathepsin G *per* neutrophil, and finally, MPO and MMP-9 *per* leukocyte in each mouse with initial instillation of LPS. In addition, we assessed the average intracellular content of proteinases for respective BAL leukocyte. We did this by lyzing BAL leukocytes and performing ELISA analysis of selected proteinases in the same groups of samples as for the assessment of extracellular proteinases *per* respective BAL leukocyte (above). For technical reasons, we then had to pool samples to calculate the *average* values of intracellular proteinase concentrations for each treatment group, thereby limiting this analysis to trends.

**Fig 6:**
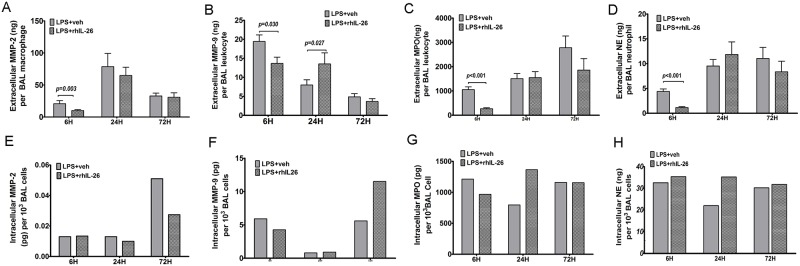
Effects of rhIL-26 on extracellular and intracellular proteinases in relation to innate effector cells in BAL samples. Mice received prior intranasal instillation of endotoxin (LPS) with or without the subsequent addition of instillation of rhIL-26 protein or its vehicle (PBS) and bronchoalveolar lavage (BAL) samples were harvested. The average contents of extracellular proteinases *per* leukocyte in BAL samples were calculated to evaluate the activity of innate inflammatory cells. The intracellular contents of proteinases were measured using ELISA in whole cell lysates of BAL samples that were pooled for each study group. The panels show **(A)** MMP-2 *per* macrophage, **(B)** MMP-9 *per* leukocyte, **(C)** MPO *per* leukocyte, **(D)** NE *per* neutrophil, **(E)** intracellular MMP-2 *per* macrophage, **(F)** intracellular MMP-9 *per* leukocyte, **(G)** intracellular MPO *per* leukocyte and **(H)** intracellular NE *per* neutrophil. Data are presented as mean ± SEM.

After prior LPS instillation, the addition of the subsequent instillation of rhIL-26 decreased the extracellular concentration of MMP-2 *per* macrophage, NE *per* neutrophil and cathepsin G *per* neutrophil, as well as the extracellular concentration of MMP-9 and MPO *per* leukocyte, as assessed at the early 6 h time-point (Fig 6A, 6B, 6C and 6D; see S3 Fig for cathepsin G). However, after prior instillation of LPS, the addition of the subsequent instillation of rhIL-26 imposed no pronounced alterations in the average intracellular MMP-2 concentration in mice at the early 6 h time-point (Fig 6E). Moreover, the same instillation of rhIL-26 caused a modest decrease of the average intracellular concentration of MMP-9 and MPO (Fig 6F, 6G and 6H). In contrast, this subsequent instillation of rhIL-26 increased the average intracellular concentration of NE at the early 6 h time-point. Likewise, the addition of the subsequent instillation of rhIL-26, after that of LPS, did increase the extracellular concentrations *per* leukocyte for MMP-9 but not for the other proteinases (Fig 6A, 6B, 6C and 6D). At the intermediate 24 h time-point after this subsequent instillation of rhIL-26, the average intracellular concentration of MMP-2 and MMP-9 remained without pronounced alterations but those of MPO and NE tended to be increased (Fig 6E, 6F, 6G and 6H). Moreover, at the late 72 h time-point the subsequent instillation of rhIL-26 caused no pronounced alterations of the extracellular concentration of each proteinase *per* respective cell type, in mice after prior instillation of LPS (Fig 6A, 6B, 6C and 6D). At this late time-point in the mice instilled with LPS followed by rhIL-26, the intracellular MMP-9 concentration in BAL leukocytes displayed a trend towards an increase whereas the other intracellular proteinase concentrations in respective leukocyte remained without pronounced alterations (Fig 6E, 6F, 6G and 6H).

## Discussion

In this study on a translational animal model that expresses the sub-units of the IL-26 receptor complex, we observed that intranasal instillation of rhIL-26 exerts multi-faceted, target-, compartment- and time- dependent effects on the innate immune response to prior instillation of LPS in the bronchoalveolar space *in vivo*. Among these effects, three stand out as a particularly important: First, there was a clear increase in the concentrations of neutrophils and macrophages in BAL samples harvested at the late 72 h time-point. Second, this effect on innate effector cells in the bronchoalveolar space was matched by a reduced inflammation score in the interstitial space, the perivascular and the peribronchial area at the same late time-point. Third, we observed that the effects caused by instillation of rhIL-26 alone were few; the most evident effects being the time-dependent effects exerted on total gelatinase activity in BAL fluid samples.

We think that it is critical that the instillation of rhIL-26 did enhance the phosphorylation of STAT3, an archetype intracellular signaling molecule downstream of the IL-26 receptor complex, in mice after theprior intranasal instillation of LPS. We demonstrated this effect at the 12 h time point after instillation(s), a finding suggesting enhanced receptor stimulation by rhIL-26 during activation of the innate immune response in the bronchoalveolar space. However, with our current evidence it is not possible to rule out that additional pathways are involved in the response to stimulation by rhIL-26. For example, further study will be required to evaluate whether, in the bronchoalveolar space *in vivo*, rhIL-26 may cause receptor signaling through TLR-9 by binding to DNA that is released by dying dendritic cells or bacteria, as was recently demonstrated by Meller *et al in vitro*, [26].

We detected mRNA for IL-10R2 and IL-20R1 in the lung tissue of our translational model, thereby verifying the gene expression for the two critical sub-units of the IL-26 receptor complex in the lungs of mice, in line with what has previously been proven in published studies [11,12,13]. This verification of the gene expression of the sub-units of the IL-26 receptor complex in mouse lungs is also in line with the previous observations that transgenic expression of human *il-26* in the lungs of mice *in vivo* does cause immunological effects and that a mouse cell line responds functionally to rhIL-26 [14]. Although we did not locate the cellular distribution of the subunits of the IL-26 receptor complex in our current study, the results from recent studies on human cells imply that IL-26 receptor subunits can be targeted by IL-26 in airway epithelial cells, bronchoalveolar leukocytes and in neutrophils [4,27].

In our translational model, the innate immune response to the initial intranasal instillation of LPS increase leukocytes (i.e. inflammatory cells) in lung tissue at all investigated time-points. Most likely, these cellular events in tissue reflected an infiltration of innate effector cells such as neutrophils and macrophages and, possibly macrophage precursor cells (i.e. monocytes). Importantly, the addition of the subsequent intranasal instillation of rhIL-26 clearly modified this innate immune response in the lung tissue. The nature of this immunomodulatory effect caused by rhIL-26 related to the specific molecular target, pulmonary compartment and time-point. At the early 6 h time-point, the referred instillation of rhIL-26 promoted the infiltration of leukocytes in lung tissue, mainly in areas surrounding the small vessels; this was most likely a sign of emigration of these cells from the circulation into the surrounding tissue. In addition, this instillation of rhIL-26 further increased macrophages in BAL samples harvested at the early 6 h time-point, most likely reflecting an accumulation of alveolar macrophages and thereby reinforcing an important immune barrier in the bronchoalveolar space. Consistent with the latter, the instillation of rhIL-26 elevated the concentrations of the macrophage chemokine CCL2 (MCP-1) in BAL samples harvested at the same time-point. Furthermore, the concentrations of CCL2 displayed a positive correlation with the concentration of macrophages in the same BAL samples. Thus, our observations imply that CCL2 induced by rhIL-26 cause an immunomodulatory effect on the accumulation of alveolar macrophages during the early phase of the innate immune response. Interestingly, the addition of the subsequent instillation of rhIL-26 tended to decrease, the average extracellular concentration of MMP-2 *per* macrophage in BAL samples at the early 6 h time-point in mice after the prior instillation of LPS. This observation is compatible with alveolar macrophages becoming less active after stimulation by rhIL-26 during the early phase of the innate immune response in the bronchoalveolar space *in vivo*, either due to a true inhibition of cellular activity in mature macrophages or due to an increased proportion of newly recruited precursor cells (i.e. monocytes), or both.

Furthermore, in line with the trend towards decreased extracellular MMP-2 per macrophage at the same early 6 h time point, the addition of the subsequent instillation of rhIL-26 decreased the extracellular concentration of MMP-9 *per* leukocyte, NE *per* neutrophil, cathepsin G *per* neutrophil in the BAL samples harvested at this time-point in mice after the prior instillation of LPS. In fact, this pattern of reduced activity was observed for MPO protein in lung tissue as well, in spite of even higher numbers of infiltrating inflammatory cells, resulting in an even more clear decrease for the assessment of MPO *per* inflammatory cell (interpreted as *per* leukocyte). Collectively, these observations support the idea that leukocytes that are recruited either in lung tissue or in the bronchoalveolar space during the early phase of innate immune response do become less active after stimulation with rhIL-26 in terms of releasing their bioactive compounds. Thus, during the early phase of the innate immune response, rhIL-26 may promote the local mobilization of macrophages into the bronchoalveolar space, while simultaneously inhibiting the activity of all innate effector cells in this compartment as well as in the surrounding lung tissue. We think that these events may serve to protect local tissue from the detrimental effects of the innate immune response but, in analogy with the reasoning for MMP-2 above, we cannot exclude that this reduced innate activity was due to the increased recruitment of precursor cells. Even more intriguing in this context, a recent study indicated that IL-26 can disrupt the epithelial barrier in the airways [28]. Whether this is also an driving factor that facilitates the movements of innate effector cells into the bronchoalveolar space is a question worthy of future study.

We think that the dynamic change of compartment-specific cellularity that was caused by rhIL-26 over time after the prior exposure to LPS in the bronchoalveolar space is important. Specifically, we found that rhIL-26 increased the number of inflammatory cells in the perivascular space of lung tissues at the early 6 h time point and in the alveolar space at the intermediate 24 as well as the late 72 h time-point. This was in sharp contrast to the reduced number of inflammatory cells in the interstitial, peribronchial and perivascular tissue compartments at the late 72 h time point after the intranasal instillation of LPS followed by rhIL-26. Taking into consideration that these chronological alterations related to different compartments, these particular findings are are compatible with the idea that rhIL-26 increases the mobilization of innate effector cells from intrapulmonary vessels towards a danger signal like LPS in the bronchoalveolar space.

In addition to the induced alterations in BAL leukocytes and cytokines, we found pronounced alterations in the mRNA levels for certain cytokines in lung tissue at matching time-points; alterations that were induced by the addition of the subsequent instillation of rhIL-26 in mice with prior instillation of LPS. For example, the stimulation with rhIL-26 increased mRNA for macrophage- (CCL2) and neutrophil-recruiting (CXCL1 and CXCL2) chemokines, as well as for a growth factor for neutrophils (G-CSF) and for more generic, pro-inflammatory cytokines (IL-6 and TNF-α); all these observations being made at the intermediate 24 h time-point mainly. However, in relation to the altered cytokine protein concentrations in BAL samples, we observed matching effects of rhIL-26 for CCL3 and IL-6 only. The locally increased transcription of CCL3 protein at this intermediate 24 h time-point may at least in part be responsible for the enhanced BAL concentrations of neutrophils later on, because we detected a strong positive correlation between these two outcomes in samples harvested at the late 72 h time-point.

Coincidentally, the recent study by Meller *et al* indicated that rhIL-26 can directly reduce the titer of bacteria in mouse lungs *in vivo*, as demonstrated at 72 h after the sequential intranasal instillation of rhIL-26 before *K*. *pneumoniae* [26]. The claim that IL-26 can bind to LPS *in vitro* [26] forwards an intriguing question, namely whether IL-26 may neutralize the effect of LPS through this binding? If this is the case, then such a binding may be relevant for immunomodulatory effect on the innate immune response to LPS that was caused by rhIL-26 in our study *in vivo*. Indeed, the reduced inflammation in lung tissue compartments that we observed after the addition of the subsequent instillation of rhIL-26 in mice after prior instillation of LPS is compatible with the proposed binding of IL-26 to LPS. However, the referred study on binding of rhIL-26 to LPS did not include any evidence that this binding actually leads to an inhibition of the biological effects of LPS. Moreover, our current study results demonstrated truly dynamic alterations of the LPS-induced innate response, representing both enhancement and inhibition, depending upon time point, lung compartment, cellular and molecular target. This makes it unlikely that a mere inhibition of the biological effects of LPS by rhIL-26 accounts for our current findings. To what extent rhIL-26 specifically interacts with other cytokines involved in the course of the innate immune response over time remains an interesting question for future study.

The evidence from our current study also forwards the possibility that rhIL-26 modifies the degradation of extracellular matrix in the lungs during the innate immune response; this is indicated by the alleviated lung tissue damage after the addition of the instillation of rhIL-26 following the prior instillation of LPS in our mouse model. When we characterized several aspects of the proteolytic activity of gelatinases and serine proteinases in BAL fluid samples, we observed that there was a decreased net proteolytic activity for both gelatinases and serine proteinases at the early 6 h time-point after the addition of the subsequent instillation of rhIL-26 following the prior instillation of LPS. This inhibitory effect caused by rhIL-26 was still present at the intermediate 24 h time-point after the instillation. Notably, we observed the inhibition of net proteinase activity in parallel with the quantitative decrease of the mentioned proteinases at the early 6 h time-point after instillation, suggesting that in this case, the reduced activity may relate to reduced quantity. The reduced quantity of proteinases, in turn, may result from decreased production in innate effector cells; an interpretation supported by the observation of a decreased average extracellular content of these proteinases *per* principal cellular source after instillation of rhIL-26 at matching time-points. However, in contrast to the evidence for decreased activation of innate effector cells at the early 6 h time point, that was caused by the addition of the subsequent instillation of rhIL-26 following the prior instillation of LPS the instillation of rhIL-26 only was not associated with any corresponding effect *per se*. At the intermediate 24 h time-point after the subsequent addition of the instillation of rhIL-26 following the prior instillation of LPS, there was still a decreased proteolytic activity of gelatinases and serine proteinases, respectively, while the quantity of these proteinases was actually increased. From a hypothetical point-of-view, this latter finding may relate to an undefined protective response in terms of anti-proteinases.

Finally, most of the immunomodulatory effects of rhIL-26 in the translational model that we utilized were modest from a quantitative point-of-view. These effects ranged from approximately 100% increase (200% of control) to a 50% reduction (50% of control) of the baseline parameters, mainly reflecting various aspects of the innate immune response. However, we think that immunomodulatory effects like the current ones may be functionally important even if with these modest magnitudes. This is because, during live bacterial infections, the enhanced release of the endogenous ligand for the IL-26 receptor complex, is likely to occur repeatedly over time and not once, as was the case in our mouse model. Moreover, it is possible that our translational lung *in vivo* model in reality illustrates sub-maximally effective effects mediated via the IL-26 receptor complex. This is because mice cannot employ endogenous IL-26 protein *per se in vivo* whereas it seems feasible that these mammals express and respond to an endogenous but currently unknown orthologue to IL-26, given the evidence for a functioning IL-26 receptor complex [11,12,13]. It is tempting to speculate that an endogenous mouse orthologue to IL-26 may compete with “the model agonist” rhIL-26 in our mouse model, thereby limiting the observed stimulatory effects on the IL-26 receptor complex.

In conclusion, the sum of integrative effects of rhIL-26 on the innate immune response to LPS in the lungs of our translational model supports that IL-26 or its species specific orthologue, may facilitate the innate immune response to a bacterial stimulus towards the bronchoalveolar space *in vivo*, presumably by stimulating the IL-26 receptor complex. At the same time, this type of cytokine signaling may contribute to the clearance of innate immune cells in th surrounding lung tissue and thereby protecting it from the detrimental effects of proteinases released by innate effector cells *in vivo*. Given that innate immune effector cells play fundamental roles in protecting the mammalian body against various kinds of infections including bacteria, this implies that targeting IL-26 receptor stimulation bears potential for treating airway infections. This target mechanism may also be of interest in chronic inflammatory airway disorders involving the innate immune response, including cystic fibrosis, COPD and certain phenotypes of asthma.

## Supporting information

S1 Fig Expression of IL-26 receptor sub-units mRNAs in lung tissues samples (related to Fig 1). Mice received intranasal instillation of recombinant human (rh) IL-26 protein or its vehicle (PBS), with or without prior instillation of bacterial endotoxin (lipopolysaccharide, LPS) or its vehicle (PBS, and were then euthanized 6 hours (h) (n = 8) and 24 h (n = 10) after the instillations. Lung tissue samples were then harvested and messenger (m)RNA was measured using RT-PCR. Data shown represents the IL-26 receptor sub-unit IL-20R1 in samples harvested 6 h **(A**) and 24 hrs **(C)** after the instillations and IL-10R2 in samples harvested 6 hrs **(B**) and 24 hrs **(D)** after the instillations. Data are presented as mean ± SEM.
(TIF)

S2 Fig Effects of rhIL-26 on BAL cytokine proteins (related to Fig 2). Mice received intranasal instillation of recombinant human (rh) IL-26 protein or its vehicle (PBS), with or without prior instillation of bacterial endotoxin (lipopolysaccharide, LPS) or its vehicle (PBS). Bronchoalveolar lavage (BAL) samples were the harvested 6 hours (h) (n = 8), 24 h (n = 10) and 72 h (n = 8) later. Cytokines concentrations were measured in the cell-free BAL fluid using Luminex^™^, including TNF-α **(A)**, KC **(B)** and MIP-2 (**C)** Data are presented as mean ± SEM.
(TIF)

S3 Fig Effects of rhIL-26 on the concentration of cathepsin G in BAL samples. Mice received intranasal instillation of bacterial endotoxin (lipopolysaccharide, LPS), with and without subsequent instillation of recombinant human (rh) IL-26 protein or its vehicle (PBS). The BAL samples were harvested at 6 h (n = 8), 24 h (n = 10) and 72 h (n = 8) after the instillations. Concentrations of cathepsin G were measured in the cell-free BAL fluid using ELISA. Data are presented as mean ± SEM.
(TIF)

S1 Table Effects of rhIL-26 on the accumulation of BAL lymphocytes. (TIF)
