# Supplementary figures and images for: Recombinant human IL-26 facilitates the innate immune response to endotoxin in the bronchoalveolar space of mice in vivo
Source: PLoS One. 2017 Dec 5;12(12):e0188909. doi: 10.1371/journal.pone.0188909 (PMC5716532; doi:10.1371/journal.pone.0188909)

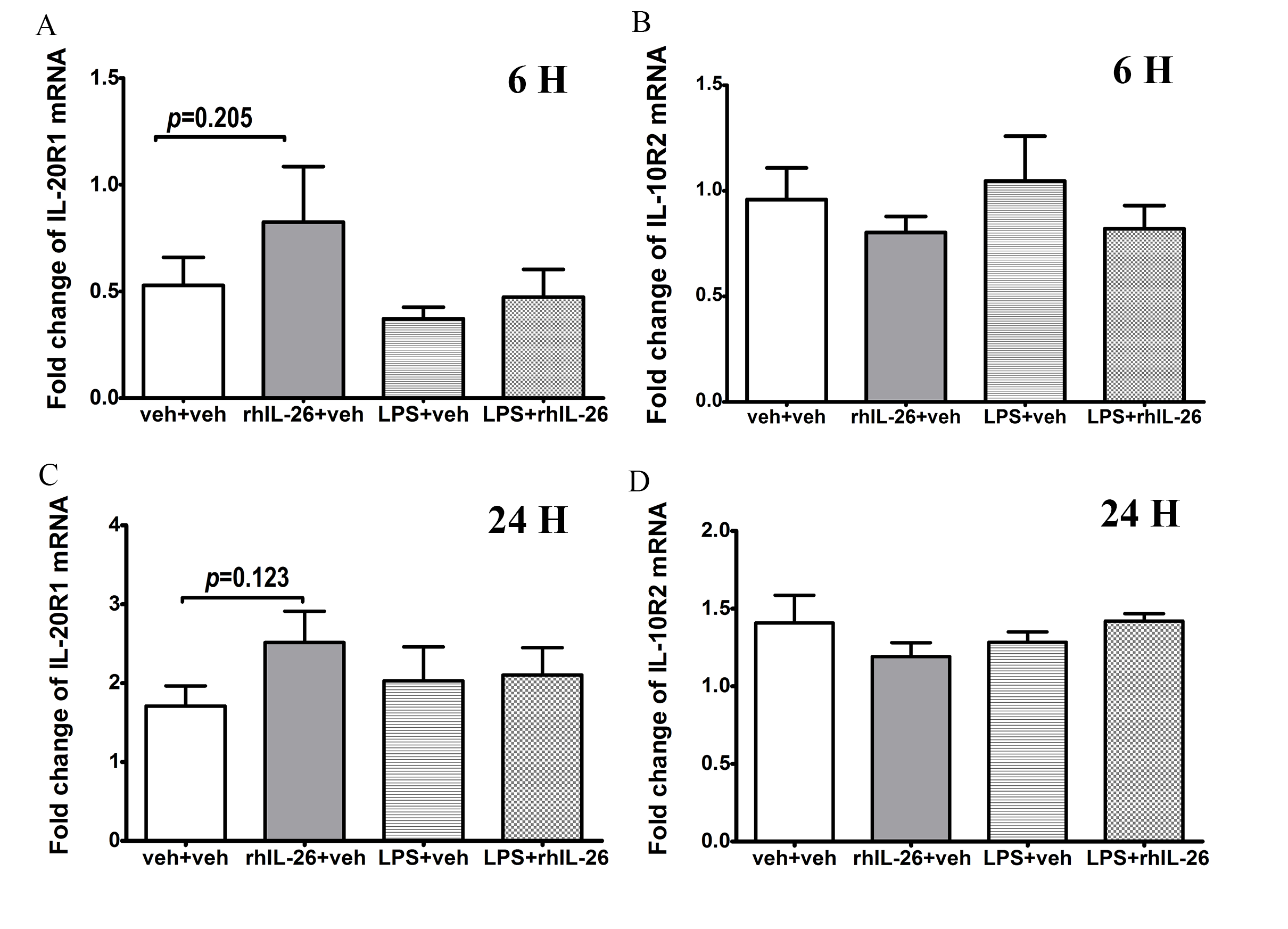

Supplement: S1 Fig — Mice received intranasal instillation of recombinant human (rh) IL-26 protein or its vehicle (PBS), with or without prior instillation of bacterial endotoxin (lipopolysaccharide, LPS) or its vehicle (PBS, and were then euthanized 6 hours (h) (n = 8) and 24 h (n = 10) after the instillations. Lung tissue samples were then harvested and messenger (m)RNA was measured using RT-PCR. Data shown represents the IL-26 receptor sub-unit IL-20R1 in samples harvested 6 h (A) and 24 hrs (C) after the instillations and IL-10R2 in samples harvested 6 hrs (B) and 24 hrs (D) after the instillations. Data are presented as mean ± SEM. (TIF) [file pone.0188909.s001.tif]

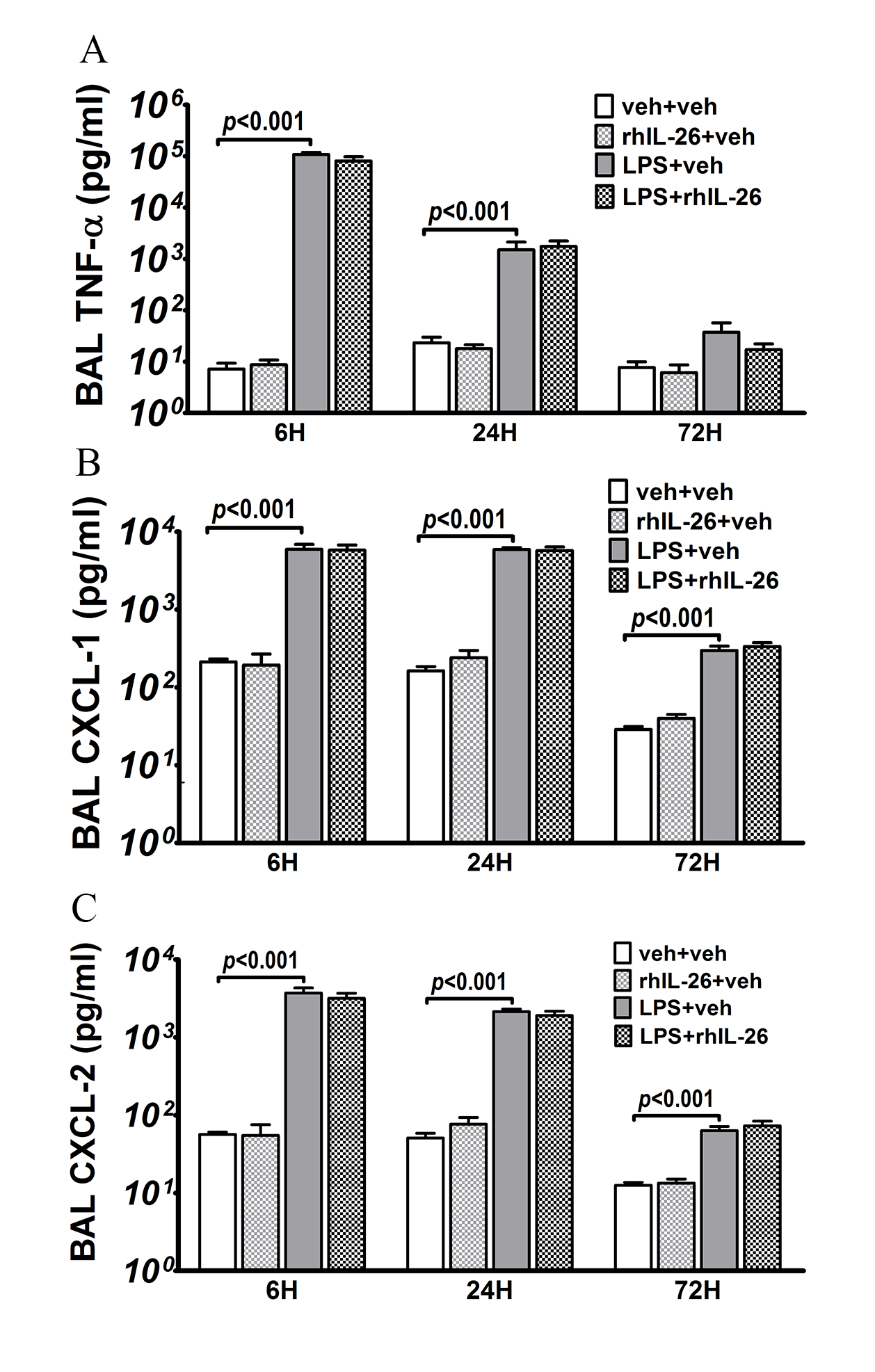

Supplement: S2 Fig — Mice received intranasal instillation of recombinant human (rh) IL-26 protein or its vehicle (PBS), with or without prior instillation of bacterial endotoxin (lipopolysaccharide, LPS) or its vehicle (PBS). Bronchoalveolar lavage (BAL) samples were the harvested 6 hours (h) (n = 8), 24 h (n = 10) and 72 h (n = 8) later. Cytokines concentrations were measured in the cell-free BAL fluid using Luminex™, including TNF-α (A), KC (B) and MIP-2 (C) Data are presented as mean ± SEM. (TIF) [file pone.0188909.s002.tif]

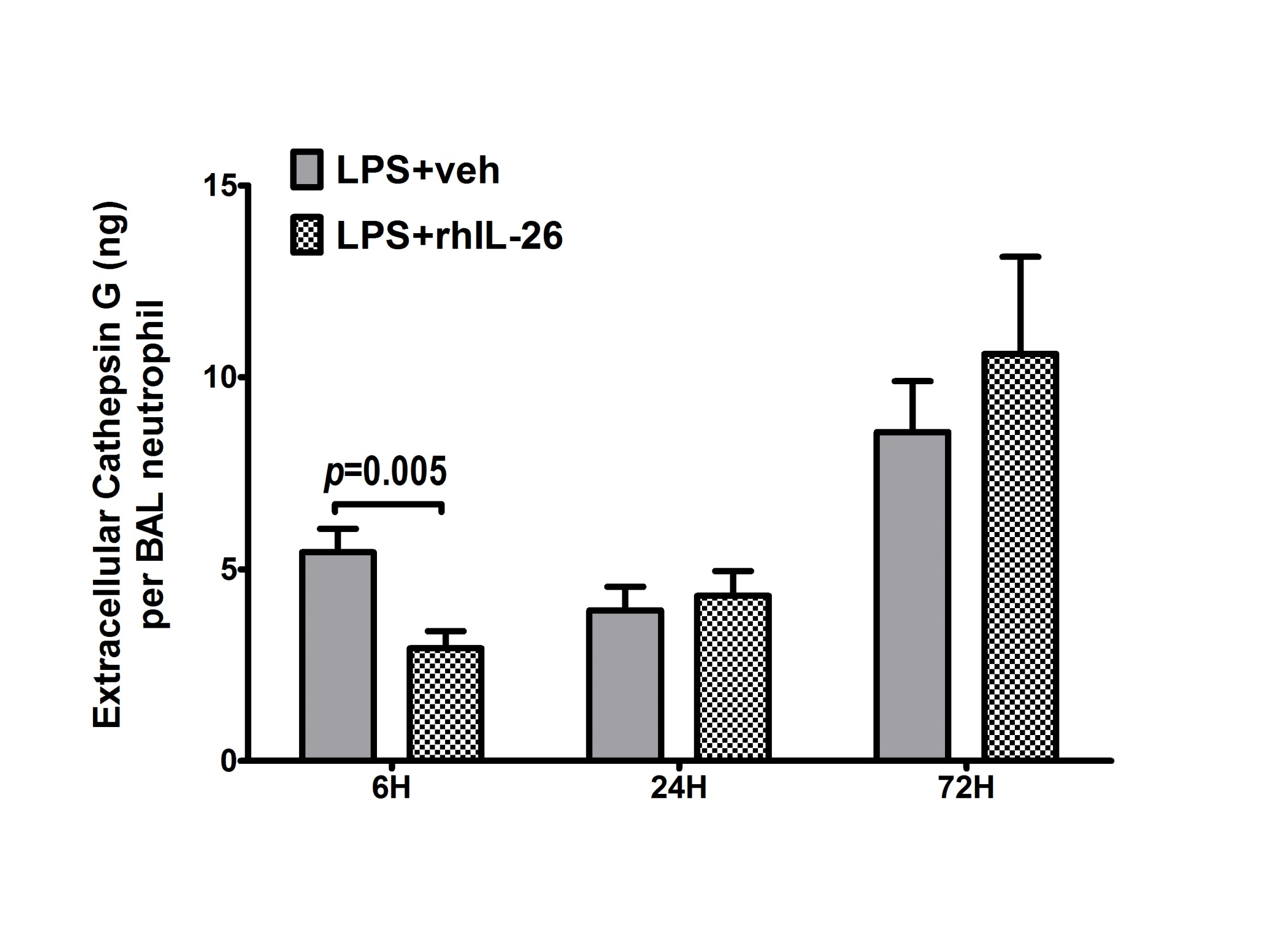

Supplement: S3 Fig — Mice received intranasal instillation of bacterial endotoxin (lipopolysaccharide, LPS), with and without subsequent instillation of recombinant human (rh) IL-26 protein or its vehicle (PBS). The BAL samples were harvested at 6 h (n = 8), 24 h (n = 10) and 72 h (n = 8) after the instillations. Concentrations of cathepsin G were measured in the cell-free BAL fluid using ELISA. Data are presented as mean ± SEM. (TIF) [file pone.0188909.s003.tif]

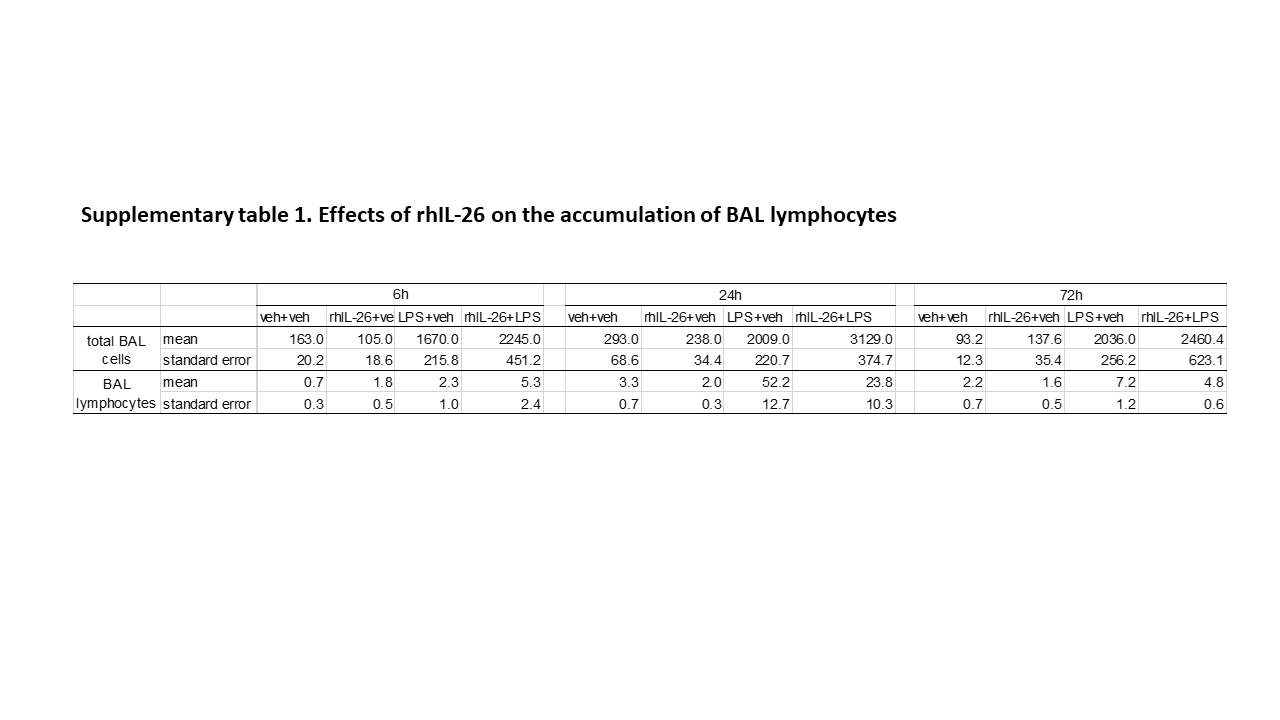

Supplement: S1 Table — (TIF) [file pone.0188909.s004.tif]
